# Freiburg Neuropathology Case Conference: Mild Disorientation and Mild Anomic Aphasia in a 79-Year-Old Female

**DOI:** 10.1007/s00062-024-01441-z

**Published:** 2024-07-29

**Authors:** A. Rau, M. Schwabenland, R. Watzlawick, M. Prinz, H. Urbach, D. Erny, C. A. Taschner

**Affiliations:** 1 Department of Neuroradiology, Medical Centre—University of Freiburg, Breisacherstraße 64, 79106 Freiburg, Germany; 2 Department of Neuropathology, Medical Centre—University of Freiburg, Freiburg, Germany; 3 Department of Neurosurgery, Medical Centre—University of Freiburg, Freiburg, Germany; 4 Faculty of Medicine, University of Freiburg, Freiburg, Germany

**Keywords:** Supratentorial ependymoma, Choroid plexus carcinoma, Intraventricular meningeoma, Intraventricular metastasis, Central neurocytoma

## Case Report

A 79-year-old female patient has been suffering from mild disorientation and mild anomic aphasia for two months. In addition, she had difficulty performing complex motor and cognitive tasks. Preoperative magnetic resonance imaging (MRI) scans showed an intraventricular lesion in the trigone of the left lateral ventricle with extension into the lateral ventricle. Subsequent surgery was performed under general anaesthesia in the prone position. The head was rotated up to 100° to the right with left shoulder elevation. After an MRI navigation-based parietotemporal craniotomy at the skull convexity, a parietal corticotomy was performed above the level of the trigone. After identification of the ventricle at 2 cm depth, the tumour presented with clear margins and slightly xantochromic red appearance. The tumour presented with hard and soft parts and was reduced in size using ultrasound aspiration. The well-demarcated tumour was circumferentially removed and intraoperative histopathological examination was performed. The preoperative neurological impairments almost completely disappeared after surgery. Complete removal of the tumour was confirmed by MRI on the first day after surgery.

## Imaging

**Fig. 1:**
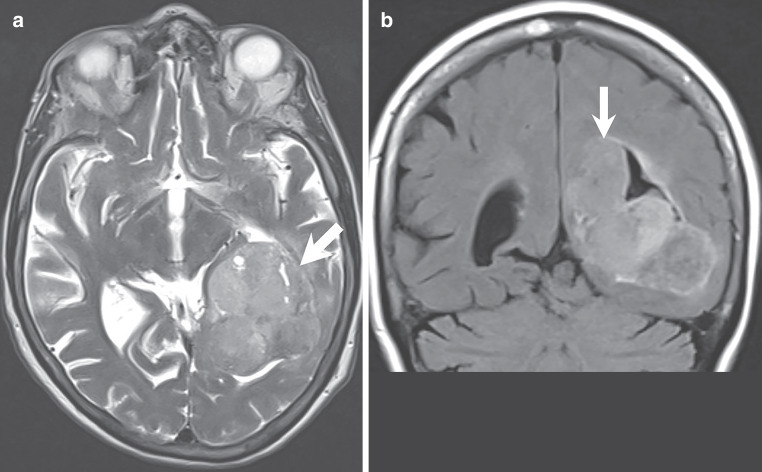
Axial T2-weighted images (**a**) and coronal FLAIR (Fluid Attenuated Inversion Recovery) images (**b**) reveal an intraventricular mass lesion (**a,** **b**, arrows) extending from the trigone (**a**) into the left lateral ventricle (**b**). The tumour matrix exhibits heterogeneous signal intensities on both T2-weighted and FLAIR images

**Fig. 2:**
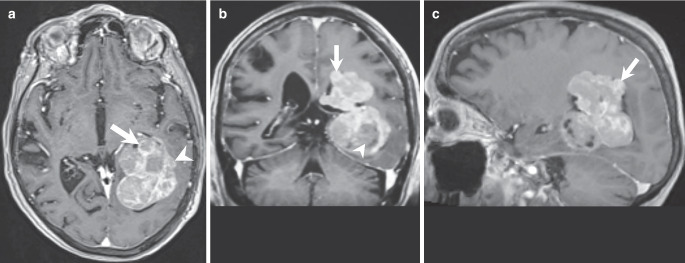
Axial (**a**), coronal (**b**), and sagittal (**c**) T1-weighted images acquired after gadolinium (Gd) administration show marked contrast enhancement of the lesion (arrows). Areas of the lesion without contrast enhancement, indicative of regressive tumour changes, are noted (**b**, arrowhead). Additionally, invasion into the surrounding brain parenchyma is evident at the lateral margin of the tumour (**a**, arrowhead)

**Fig. 3:**
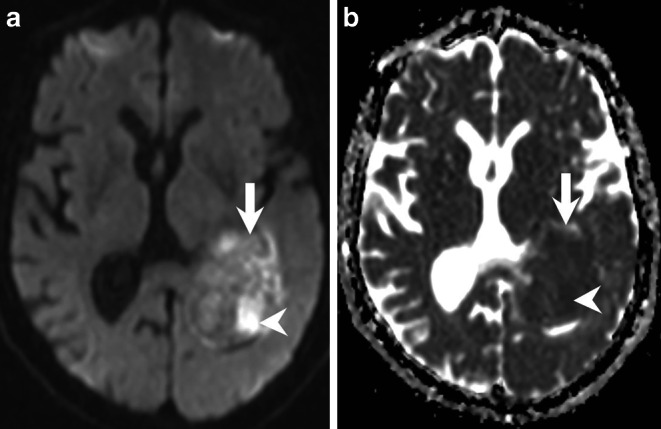
Axial diffusion-weighted images (b-value: 1000) (**a**) and apparent diffusion coefficient maps (**b**) display diffusion restriction within the solid, contrast-enhancing parts of the lesion (**a,** **b**, arrows), suggesting hypercellular tumour compartments

Magnetic resonance imaging upon admission revealed a large intraventricular mass lesion extending from the trigone up to the left lateral ventricle. The tumour matrix exhibited heterogeneous signal intensities on both T2-weighted and FLAIR images (Fig. 1a, b, arrows). T1-weighted images acquired after gadolinium (Gd) administration showed marked contrast enhancement of the lesion (Fig. 2a–c, arrows). Areas of the lesion without contrast enhancement, indicative of regressive tumour changes, were noted (Fig. 2b, arrowhead). Additionally, invasion into the surrounding brain parenchyma was evident at the lateral margin of the tumour (Fig. 2a, arrowhead). Diffusion-weighted images displayed diffusion restriction within the solid, contrast-enhancing parts of the lesion (Fig. 3a, b, arrows), suggesting hypercellular tumour compartments.

## Differential Diagnosis

In general, most intraventricular neoplasms have no specific clinical symptoms, but rather present with signs of increased intracranial pressure or obstructive hydrocephalus such as headache, seizures, and altered mental status. Imaging may reveal circumscribed or diffuse leakage of cerebrospinal fluid (CSF) into the brain parenchyma, depending on the localization.

### Supratentorial Ependymoma

Supratentorial ependymomas arise from the ependymal lining of the ventricles of the brain and comprise distinct molecular features in comparison with spinal ependymomas. The 2021 WHO classification describes two subgroups characterized by either fusions on chromosome 11 involving the coactivator RELA (poor prognosis, more frequent in older children) or YAP1 fusions (better prognosis, more common in patients younger than 3 years). While this in general uncommon tumour typically occurs in children, a small peak in the 2nd to 3rd decade of life with a male predisposition was described [1]. Ependymomas are mostly located in the posterior fossa, while one third occurs supratentorially. An intraventricular localisation is less common than a parenchymal one. The mass itself is frequently well-delineated and lobulated with cystic and calcified components. Hemorrhagic portions can be found in a small proportion. In CT, large mixed hypo- to isoattenuation of the solid part is found. Calcifications range from punctuate to very extensive. On MRI, ependymomas appear isointense to slightly hypointense on T1-weighted images and isointense to hyperintense on T2-weighted images. Enhancement after contrast administration is typically heterogeneous [2].

Although the imaging features of our case were compatible with an intraventricular ependymoma, we considered it less likely as ependymomas tend to occur in pediatric patients and are rarely seen in the lateral ventricles.

### Choroid Plexus Tumours

Choroid plexus tumours comprise choroid plexus papilloma (CCP; WHO grade 1), atypical choroid plexus papilloma (WHO grade 2) and choroid plexus carcinoma (CPCa; WHO grade 3) [1]. CCP are rare and constitute 0.5% of brain tumours in adulthood and 2–3% of pediatric brain tumours. While in adults, they usually occur in the fourth ventricle, localisation in the lateral ventricle is almost exclusive to pediatric patients. CPCa almost exclusively occurs in young children and represent 20–40% of choroid plexus tumours [2]. Choroid plexus tumours have a substantial overlap in imaging features and cannot reliably be distinguished on imaging alone [3]. Imaging reveals multilobulated to cauliflower-like tumours which are mostly well-delineated. They are iso- to hyperdense on non-contrast CT and calcifications are present in about 25% of cases. On MRI, a heterogeneously isointense to hypointense signal is typically observed on T1w with isointense to hyperintense signal on T2w. Most CPP and CPCa show strong, homogeneous enhancement. Heterogeneous enhancement, extensive brain invasion or necrosis may indicate a higher-grade tumour [3].

Localization in the lateral ventricle is not typical of CCP in an adult. Although CPCa shows heterogeneous contrast uptake and is mainly localized in the lateral ventricle, this was unlikely in our case of an elderly patient [4]. Consequently, we did not consider it a valid differential diagnosis.

### Intraventricular Meningioma

Intraventricular meningioma account for only 0.5–3% of all meningiomas, and have a peak incidence around the age of 50, whereas they are rare in childhood [5].

As dural contact is only present in a subset of intraventricular menigiomas, the exact pathophysiology remains elusive. However, the origin of meningothelial inclusion bodies in the choroid plexus is frequently suggested [2, 5].

The most frequent localisation of intraventricular meningioma is in the trigone of the lateral ventricle (> 80%) [5]. Imaging of patients with intraventricular meningiomas show extra-axial, well-circumscribed masses that share most of the imaging features of typical meningiomas. They show predominantly hyperdense (70%) to isodense (30%) attenuation on CT and are slightly hypointense to the cortex on T1w MRI. In contrast, the T2w signal is variable, depending on histopathology and fibrous components. In general, homogeneous contrast is observed, which may be more heterogeneous in higher WHO grades [6].

In our case, the trigonal location of the lesion favors the diagnosis of intraventricular meningioma, while the heterogeneous contrast enhancement pattern is rather unusual.

### Intraventricular Metastasis

In general, intraventricular metastases are infrequent, but are most commonly found within the lateral ventricles adjacent to the choroid plexus [7]. Kidney cancer is known to be the most frequent origin, followed by thyroid, lung, colon, melanoma and breast malignancy [7, 8]. Imaging of a solitary choroid plexus metastasis cannot reliably differentiate from meningioma or primary choroid plexus tumour [2].

Although our patient had no history of malignancy, we could not rule out solitary metastatic disease of the left choroid plexus in an elderly woman.

### Central Neurocytoma

Central neurocytomas are WHO grade 2 neuroepithelial intraventricular tumours, accounting for < 1% of intracranial tumours [1]. This entity is mostly seen in young adults with a mean age at diagnosis of 20–40 years. As they are arising from the septum pellucidum, central neurocytomas are typically located within the lateral ventricles in the vicinity of the foramen of Monro [9].

Imaging reveals well-circumscribed intraventricular masses and both micro- and macrocystic components are frequent leading to a rather heterogeneous appearence, escpecially in larger tumours [10]. On CT images, the solid component is hyperattenuating in comparison with adjacent white matter and with punctate calcifications present in > 50% [9]. MRI shows a heterogeneous signal which is isointense on T1w and hyperintense on T2w. T2* or susceptibility weighted imaging (SWI) frequently reveals calcifications, too. Contrast enhancement is typically moderate to strong in central neurocytoma [2].

Although we observed a multilobulated mass with heterogeneous contrast uptake in the lateral ventricle in the present case, the lack of contact with the septum pellucidum and the trigonal localization made the diagnosis of central neurocytoma less likely.

## Histology and Immunohistochemistry

**Fig. 4:**
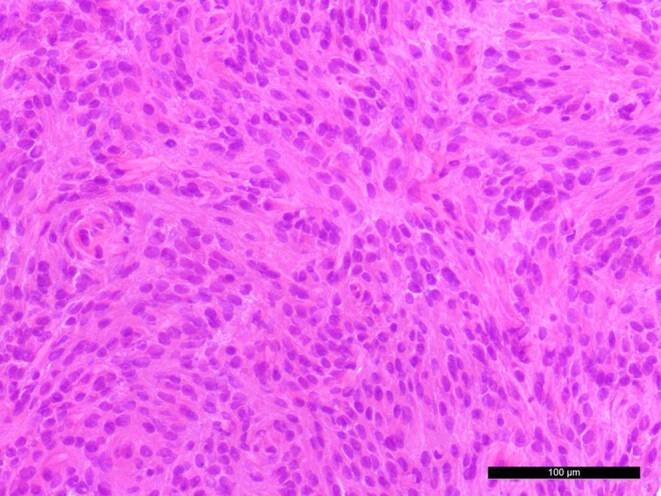
An intraoperative cryostat section stained with hematoxylin and eosin (H&E) showed an isomorphic tumour with round and elongated nuclei. Scale bar: 100 µm

A biopsy of the intraventricular mass was obtained for intraoperative neuropathological examination. The hematoxylin and eosin (H&E) stained cryostat section showed an isomorphic tumour (Fig. 4). The tumour cells were characterized by round and occasionally elongated nuclei. Nuclear holes (internal empty spaces) were observed. Therefore, a meningioma was suspected. After the surgical resection, additional tumour tissue was fixed in formaldehyde and embedded in paraffin (FFPE).

**Fig. 5:**
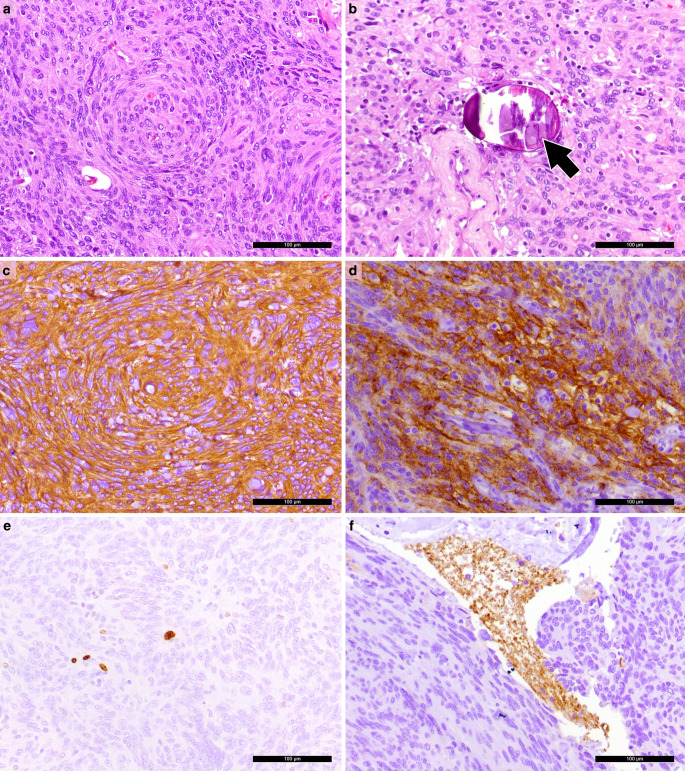
**a** The H&E stained formalin-fixed paraffin-embedded (FFPE) section showed a tumour with a syncytium-like appearance with tumour cells being arranged in lobules/whorls. Nuclear holes could be observed. **b** Psammoma bodies (arrow) were detected in the H&E stained section. **c** The immunohistochemical reaction for Vimentin (brown) was positive. **d** Positivity was also observed in the immunohistochemical reaction for epithelial membrane antigen (EMA, brown). **e** Mitotic activity was low, as indicated by the immunohistochemistry for Ki-67 (MIB‑1, brown). **f** Synaptophysin-positive (brown) central nervous system tissue was found within the tumour mass, indicating brain invasion. Hematoxylin (blue) was used as counterstaining for immunohistochemical reactions. Scale bars **a–f**: 100 µm

The H&E stained FFPE section confirmed the findings of the intraoperative examination. The isomorphic tumour had a syncytium-like appearance. The tumour cells displayed clear nuclear holes and were occasionally arranged in lobules (Fig. 5a). Psammoma bodies, a characteristic feature of meningiomas, were detected, albeit rarely (Fig. 5b).

Immunohistochemical reactions were performed to further characterize the tumour. The tumour cells showed strong positivity for vimentin (Fig. 5c) and epithelial membrane antigen (EMA, Fig. 5d). The proliferation marker MIB‑1 (Ki-67) highlighted up to 2% of tumour cells, indicating a low proliferation index (Fig. 5e). Mitotic activity was low, with two mitotic figures identified in 10 high-power fields (HPF).

Additionally, Synaptophysin immunohistochemistry revealed a small cluster of CNS tissue entrapped within the meningioma, indicating brain invasion (Fig. 5f). Due to this brain invasion and despite the low mitotic activity, the tumour was classified as atypical meningioma, CNS WHO grade 2.

## Diagnosis

### Atypical meningioma, CNS WHO grade 2

The fifth edition of the WHO classification of central nervous system tumours defines atypical meningioma as a meningioma with certain atypical features, including increased mitotic activity or evidence of brain invasion, that do not meet the criteria for anaplastic (malignant) meningioma [11]. Atypical meningiomas are graded as CNS WHO grade 2. These tumours are characterized by a higher likelihood of recurrence and more aggressive behaviour compared to CNS WHO grade 1 meningiomas [11].

Meningiomas are thought to originate from the arachnoid cap cells of the meninges. They can occur in various locations within the central nervous system but are commonly found along the convexities of the brain, the falx cerebri, and the base of the skull. An intraventricular localization is a rare, accounting for 0.5–3% of all meningiomas [5]. The majority of intraventricular meningiomas are located in the lateral ventricles (88.4%), with a smaller proportion found in the fourth ventricle (8.7%) and the third ventricle (2.9%) [5]. Arachnoid cells, which may be present in the choroid plexus, are believed to be the origin of intraventricular meningiomas [12].
